# Bubble coalescence and dissolution effects on sonochemical activity in pulsed wave ultrasound systems^☆^

**DOI:** 10.1016/j.ultsonch.2026.107787

**Published:** 2026-02-16

**Authors:** Marc C. Nolan, Haleigh A. Fernandez, Amanda N. Cowen, Fallon R. Fuller, Linda K. Weavers

**Affiliations:** a Environmental Sciences Graduate Program, The Ohio State University, Columbus, OH 43210, USA; b Department of Civil, Environmental, and Geodetic Engineering, The Ohio State University, Columbus, OH 43210, USA; c Ohio Water Resources Center, The Ohio State University, Columbus, OH 43210, USA

**Keywords:** Pulsed Wave Ultrasound, Bubble coalescence, Bubble dissolution, Calorimetry, Sonochemical activity, Fluorotelomer sulfonates

## Abstract

Pulsed wave (PW) ultrasound offers a promising alternative to continuous wave (CW) ultrasound for contaminant degradation, yet the mechanisms governing PW sonochemical effectiveness remains underexplored. This study investigates the relative influence of bubble coalescence and dissolution on sonochemical activity in PW systems using calorimetry, terephthalate (TA) dosimetry, and the capillary method. Experiments were conducted at 337 kHz with 10 and 100 ms on-times with varying off-times and surfactant concentrations using fluorotelomer sulfonates (FtS) of differing chain lengths. Calorimetry and hydroxyterephthalate (HTA) fluorescence consistently showed less sonochemical activity with increasing off-times. However, bubble coalescence, measured by the capillary method, decreased with increasing off-times. These findings, taken together, indicate that bubble dissolution, rather than coalescence, is the dominant factor limiting sonochemical efficiency in PW systems. In the presence of FtS and compared to CW systems, PW did not affect bubble coalescence, suggesting surfactants affect bubble coalescence similarly in CW and PW systems. Finally, calorimetry proved to be a reliable, chemical-free dosimeter for assessing cavitation activity, offering a practical alternative to traditional dosimeters.

## Highlights

• Calorimetry is a reliable, chemical-free sonochemical dosimeter.
• Sonochemical activity declines as the ultrasound off-time increases.
• Longer on-times increase sonochemical activity compared to shorter ones.
• Bubble dissolution controls sonochemical effectiveness in PW systems.

## 1 Introduction

Sonolysis utilizes high power soundwaves between the frequencies of 16 – 1000 kHz to achieve contaminant degradation via acoustic cavitation [1], [2], [3], [4], [5], [6], [7]. During sonication, bubbles are formed from preexisting gas cavities in solution and continue to grow through a process known as rectified diffusion. During rectified diffusion, bubbles undergo numerous cycles of expansion (rarefaction) and contraction (compression) [8], [9], [10], [11], [12]. Bubbles become sonochemically active if they overcome the Blake threshold pressure. Bubbles that overcome this threshold pressure produce sonochemical effects through cavitation [13]. During cavitation, bubbles collapse adiabatically compressing and heating the gases within the bubbles creating what is known as a “hot spot.” These gaseous hot spots are localized areas of high temperatures and pressures (5000 K and 1000 bar) that result in thermolytic degradation of volatile and surface-active contaminants that accumulate inside and at the air–water interface, respectively [1], [4], [6], [7], [14]. While ultrasound has been shown to degrade numerous contaminants, there has been little investigation into how pulsed wave (PW) ultrasound influences bubble dynamics and sonochemical activity.

In contrast to continuous wave (CW) ultrasound, PW ultrasound has pulse intervals, the “off” times of no ultrasound exposure, in between the pulse lengths, or the “on” times of ultrasound exposure. The introduction of off-times increases the lifetime of cavitation bubbles, allowing more time for surface-active compounds to partition to bubble interfaces [15]. This additional time allows for increased accumulation of surface-active compounds at the bubble interface leading to increased degradation. Yang et al. 2005 [16] observed decreased sonochemical degradation of surfactants with shorter pulse on-times, but also that extended off-times enhanced sonochemical degradation at higher concentrations of surfactants. They attributed enhanced degradation rates to higher surface accumulation of surface-active compounds with longer off-times. They also determined that surface activity and concentration of the solute, as well as the on- and off-times play an important role in the efficiency of PW systems [16], [17], [18], [19]. In addition, PW ultrasound has been shown to enhance the sonochemical efficiency due to increasing the spatial distribution of soundwaves. [20], [21] This alteration in the acoustic field leads to an increase in active bubbles and therefore sonochemical activity.

Henglein et al. 1989 [22] describes the chemical activity of pulsed systems in regard to “activation” and “deactivation” times. Sonochemical systems require a minimum number of acoustic cycles necessary to “activate” the system, or time to produce cavitating bubbles. Clarke et al. 1970 [23] found that the optimal number of cycles for acoustic systems lies between 10^4^ and 10^5^ cycles. The activation time is determined by the on-time. The on-time must be long enough to create a bubble that is large enough to withstand the pressures exerted upon it during the off-times. The deactivation time is the time it takes the bubbles created during the pulses of ultrasound exposure to no longer be sonochemically active. If the off-time is longer than the deactivation time, the residual pressure amplitude created from the previous pulse cannot sustain cavitation bubbles [20]. The cavitation bubbles in this scenario will either coalesce or dissolve causing a decrease in cavitating bubbles. This results in the system having to reactivate during the next pulse on-time. Large amounts of bubble coalescence and/or dissolution lead to decreases in active bubbles, therefore decreasing the sonochemical activity of the system.

Bubble coalescence occurs in the absence and presence of ultrasound. In the absence of ultrasound, gas bubbles move toward one another due to the hydrodynamic forces of the liquid [24], [25]. When two bubbles come in contact, a thin film forms between them. The bubbles will coalesce if they remain in contact long enough for the thin film to rupture [26], [27].

Upon applying an ultrasonic field, the amount of bubble coalescence in liquids significantly increases and the steps for coalescence become more complicated. Bubble coalescence occurs in an acoustic field due to both primary and secondary Bjerknes forces. The primary Bjerknes force moves bubbles to the nodes and antinodes. Once the bubbles have been collected at the nodes or antinodes, the secondary Bjerknes forces cause bubbles that are oscillating in phase and of similar size to attract and coalesce [26], [27], [28]. Depending on their size, coalesced bubbles either grow to an active size and cavitate or become “degassing” bubbles. Degassing bubbles have radii that are greater than the range for cavitation and exit solution due to buoyant forces. An increase in degassing bubbles leads to a decrease in sonochemical activity through decreasing the number of active bubbles [10], [20], [29]. During the silent periods in PW systems, however, there are no ultrasound waves to drive bubbles to the nodes and antinodes. While coalescence may occur in PW systems, evidence suggests [18] it is less prevalent than in CW systems.

Bubble dissolution is thought to increase in PW systems due to the off–times in ultrasound exposure. Sonoluminescence has been used to measure sonochemical activity in PW systems in comparison to CW systems [29], [30], [31]. These studies determined that longer on-times led to increased sonoluminescent intensities. Similarly, Brayman et al. 1996 [32], 1997 [33], and 1999 [34] utilized inertial cavitation (IC) detection and observed that inertial activity is dependent upon the on-times of ultrasound exposure. Too short an on–time prevents bubbles from growing enough to withstand Laplace pressure, leading to dissolution during off–times [15], [17], [22], [35]. Therefore, coalescence and dissolution play important roles in determining the effectiveness of PW systems and determining parameters that minimize both processes is key to optimizing degradation in PW systems.

The purpose of the present study is to determine whether bubble coalescence or dissolution plays a more important role in sonochemical effectiveness in PW systems. Calorimetry was used as a simple, chemical-free method to observe sonochemical activity, an alternative to traditional dosimeters. Additionally, calorimetry was used to probe the role of solutes on sonochemical activity. Terephthalate oxidation was used as a dosimeter to validate the use of calorimetry to monitor sonochemical activity. The capillary method was employed to measure bubble coalescence. The results from the three methods allowed for a more thorough understanding of how coalescence and dissolution affect sonochemical reactions in PW systems. Various on– and off-times were studied to determine the effects of pulsing parameters on sonochemical activity. Surfactants with varying concentrations and surface activities were used to study the effects of surface accumulation on bubble coalescence and dissolution.

## 2 Materials and methods

### 2.1 Materials

LCMS grade (99.8%) methanol used for cleaning glassware was purchased from Fisher Scientific. 1H,1H,2H,2H-Perfluorohexanesulfonic acid (4:2 Fluorotelomer sulfonic acid (4:2 FtS)), 1H,1H,2H,2H-Perfluorooctanesulfonic acid (6:2 Fluorotelomer sulfonic acid (6:2 FtS)), and 1H,1H,2H,2H-Perfluorodecanesulfonic acid (8:2 Fluorotelomer sulfonic acid (8:2 FtS)) salts were purchased from SynQuest Laboratories. The condensed structural formulas for 4:2, 6:2, and 8:2 FtS are CF_3_(CF_2_)_3_(CH_2_)_2_SO_3_H, CF_3_(CF_2_)_5_(CH_2_)_2_SO_3_H, and CF_3_(CF_2_)_7_(CH_2_)_2_SO_3_H, respectively. Argon (99.998%) gas cylinders used for sparging solutions were obtained from Linde Gas & Equipment Inc. Deionized (DI) ultra-pure, filtered water (MilliQ Millipore) was used for making all solutions.

### 2.2 Ultrasound and experimental conditions

The ultrasonic waves were produced via a 337 kHz flat-plate transducer (Meinhardt ultrasound transducer E 805/T/M) with an irradiation area of 17.35 cm^2^. The flat plate transducer was connected to a Meinhardt ultrasound multifrequency power generator that supplied continuous or pulsed wave signals operating at 100% amplitude. The total experimental time of the PW experiments differed due to the on:off ratios. Equation (1) below was used to adjust the total experiment time to ensure equal sonication time across all PW conditions and for comparison to CW ultrasound.

(1) $$t_{\mathrm{total}}=t_{\mathrm{sonication}}*\left(1+1/\left(t_{\mathrm{on}}/t_{\mathrm{off}}\right)\right)$$

where t_total_ is the total experimental time including the on– and off-times, t_sonication_ is the total time ultrasound is on (on-time), t_o__n_ is the duration of an ultrasound pulse (summation of on-times), and t_off_ is a silent period between ultrasound pulses (off-time).

Experiments in DI water under CW ultrasound were conducted for all methods as base measurements. Various on– and off-times were used to observe how pulsing affects sonochemical output. In addition, various concentrations (0.1, 1, 10, and 100 µM) and chain lengths of fluorotelomer sulfonates (4:2, 6:2, and 8:2 FtS) under CW and PW settings probed the role surface accumulation and surface activity of contaminants play in bubble activity.

#### 2.2.1 Calorimetry experiments

Following Contamine et al. 1995 [36], calorimetry experiments were conducted to determine the amount of power (W) delivered to the system. Fig. S1 in the Supplementary Information (SI) shows the experimental setup used for calorimetry experiments. A 250 mL solution was placed inside a 300 mL double-walled, glass reactor. Experiments were run with air and no buffer. Control experiments with argon sparge showed no difference in calorimetric power (Table S1). All calorimetry experiments were conducted with a t_sonication_ of 4.5 min. The change in temperature was monitored using an Omega HH12B thermocouple meter with type K thermocouple. The starting temperature of the solutions was approximately 20 °C, and the final temperature after sonication was generally between 29 and 32 °C.

Heat loss to the surroundings was minimized. As shown in Fig. S1, a type T thermocouple, to monitor the heat transferred from the liquid to the surroundings, was placed inside the air-filled cooling jacket and in contact with the reactor wall containing the reacting solution. The type T thermocouple was used to monitor potential heat loss from the system during experiments. The amount of heat transferred to the surroundings during PW experiments was not statistically different compared to CW experiments (Table S2).

Equation (2) was used to calculate the Power (W) delivered to solution:

(2) $$Power\;\left(W\right)=mC_{p}\left(dT/dt\right)$$

where m is the mass of the solution (g), C_p_ is the heat capacity of water (J/gK), and dT/dt is the slope of the linear regression obtained by graphing the change in temperature (T) over time (t).

#### 2.2.2 Fluorescence measurements of 2-Hydroxytereplthate (HTA)

**Scheme 1:**
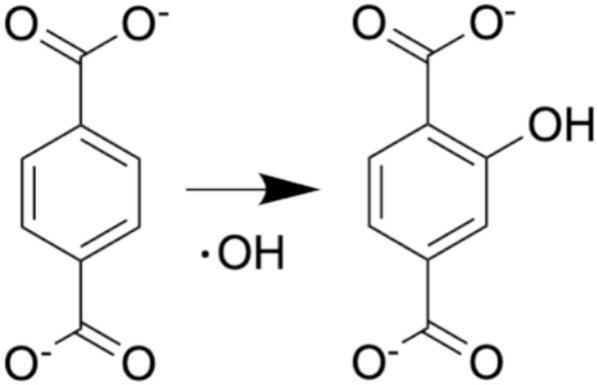
Oxidation reaction between TA and hydroxyl radical to form HTA. Carboxylic acids are shown in the deprotonated form because of the pH of all experiments.

Following Price et al. 1993 [37], hydroxyl radical production from cavitating bubbles was estimated through fluorescence measurements of 2-hydroxytereplthalic acid (HTA) formation. 200 mL of 1 mM terephthalic acid buffered with 25 mM of NaH_2_PO_4_ and Na_2_HPO_4_ was placed in the same reactor as calorimetry experiments. The cooling jacket (set to 10 °C) was used to maintain stable solution temperature. The pH of the solutions remained between 7.0 and 7.5 to ensure terephthalic acid was in its fully deprotonated form terephthalate. Scheme 1 shows the reaction of hydroxyl radicals, produced from sonolysis of water, and TA producing fluorescent HTA. The solution was sparged with argon gas for 10 min before sonication and the argon gas was continuously pumped into the headspace of the reactor. All HTA oxidation experiments were conducted with a t_sonication_ of 15 min with aliquots taken at various time points.

An Agilent Cary Eclipse Fluorescence Spectrophotometer was used to detect fluorescence from HTA formation. The excitation wavelength was 315 nm and emission wavelength set to 426.06 nm. The excitation and emission slit widths were set to 5 and 2.5 nm, respectively.

#### 2.2.3 Coalescence measurements

Following Lee et al. 2005 [28], the capillary method, correlating to the amount of bubble coalescence, was used to measure the change in total bubble volume (ΔV_T_) during ultrasound experiments. Approximately 130 mL of solution was placed in a glass capillary cell (Fig. S2) and sparged with argon gas for five minutes. The solution was allowed to rest for a few minutes to allow gas bubbles in solution to reach an equilibrium. Once the capillary tube was placed in the solution, a small amount of solution was drawn into the tube due to the initial capillary force. The capillary tube was then fastened to the neck of the capillary cell. The water level inside the tube increased slightly due to the increase in pressure from the seal. The post–sealing equilibrium water level served as the initial height measurement.

All capillary experiments were conducted with a t_sonication_ of 10 s. Once ultrasound exposure ceased, the water level dropped slightly due to the dissolution of uncoalesced bubbles that are smaller than the range of active bubbles. In both CW and PW, after approximately ten seconds, the water level stabilized and was used as the final height measurement. The change in volume height in the capillary tube was used to determine the total change in bubble volume (ΔV_T_).

During PW experiments, ΔV_T_ fluctuated: it increased during each on–time and decreased during each off–time. The water–level drop during off–times was noticeable but smaller than the decrease that occurred as the capillary level stabilized at the end of the CW and PW experiments. Control experiments indicated a negligible amount of temperature change in the experiments (Table S3); therefore, a cooling bath was not used to maintain temperature. Select experiments did not show a change in pH (Table S4) in the ten seconds of sonication; therefore, pH was not monitored for each experiment.

#### 2.2.4 Statistical analysis of data

Reported error and error bars indicate the standard error from triplicate measurements.

## 3 Results and Discussion

Off-times with 10 and 100 ms on-times were studied to determine the effect of pulsing parameters on bubble coalescence and dissolution. Off-times were selected to have the same on:off ratios for the 10 and 100 ms on-times. The on-times selected for investigation fall inside and outside of the optimal range of 10^4^ and 10^5^ cycles necessary to activate an acoustic system [23]. On-times of 10 and 100 ms for our study were chosen because they correspond to 3.37 x 10^3^ (outside optimal range) and 3.37 x 10^4^ cycles (inside the optimal range), respectively. Henglein et al. 1990 [38] and Gutierrez et al. 1990 [35] found that on-times corresponding to 1.0 x 10^4^ and 1.0 x 10^5^ cycles per pulse produced the highest iodine yields and polymer degradation rates in comparison to 2.0 x 10^3^, 5.0 x 10^3^, and 1.0 x 10^6^ cycles. In addition, Yang et al. 2005 [16] found that pulse on-times of 1.77 x 10^4^, 3.54 x 10^4^, and 7.08 x 10^4^ cycles produced the highest enhancement of PW degradation of the surfactant 4-octylbenzene sulfonate (OBS).

### 3.1 Using calorimetric power as a dosimeter in PW systems

Early attempts to characterize sonochemical activity in PW fields employed dosimetry techniques such as luminol and potassium iodide oxidations [22], [35], [38]. While these methods infer the relative number of active bubbles from hydroxyl radical production, it cannot be determined whether changes in the sonochemical activity of the system are due to bubble dissolution or coalescence. In addition, these dosimeters cannot be used to determine changes to sonochemical activity in the presence of compounds that interact with hydroxyl radical. We explored the use of calorimetry as an easier, chemical-free dosimeter. The rate of change in temperature of a solution being sonicated corresponds to the heat released from actively collapsing cavitation bubbles in the solution [36]. More and/or hotter cavitating bubbles correspond to higher calorimetric powers, and vice versa. The PW conditions tested focused on the extended off-times to probe how sonochemical activity is affected by the silent periods between ultrasound pulses.

**Fig. 1:**
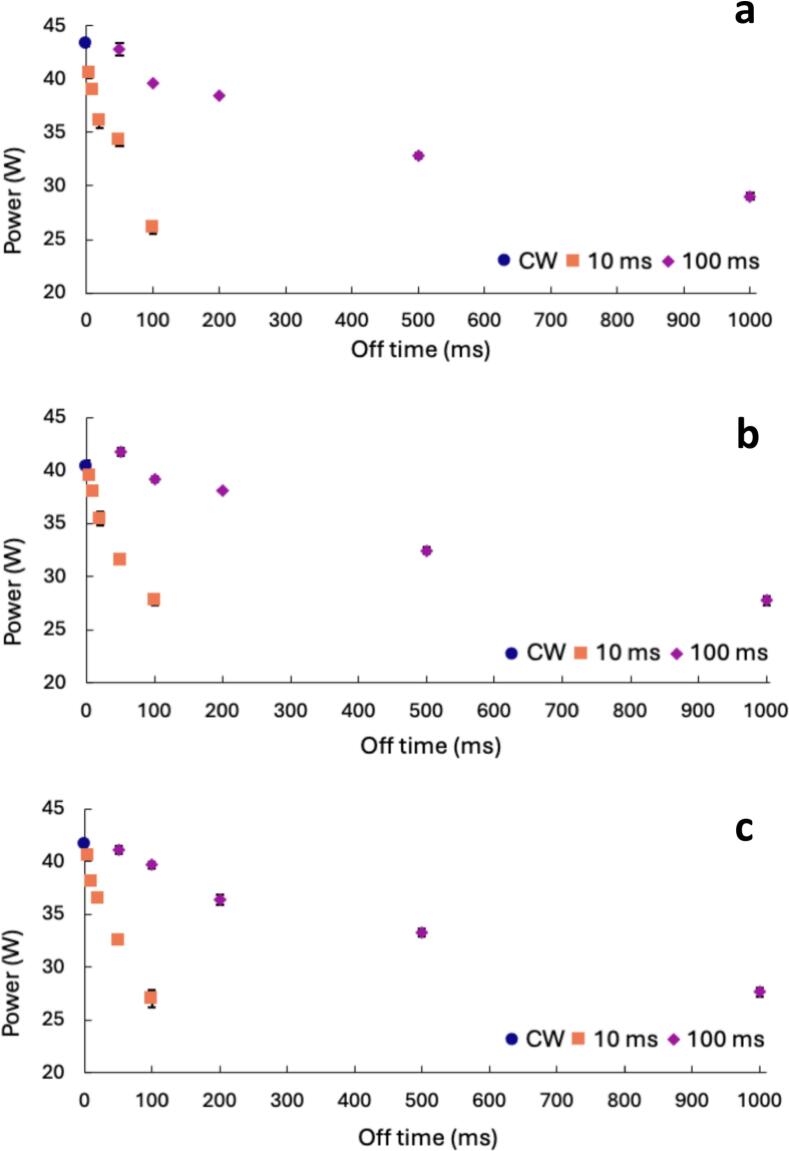
Calorimetric power determined under CW (blue circle), 10 ms on-time (orange square), and 100 ms on-time (magenta diamond) with on:off ratios from 1:0.5 to 1:10 in a) DI water, b) 100 µM of 4:2 FtS, and c) 100 µM of 8:2 FtS at a frequency of 337 kHz. Error bars indicate standard error from triplicate experiments. In some cases, error bars are covered by the symbol.

Fig. 1 and Table S5 – S7 in the SI show calorimetry results under PW conditions in DI water, 100 µM of 4:2 FtS, and 100 µM of 8:2 FtS. In DW water (Fig. 1a), calorimetric power decreased under all PW conditions relative to CW, except for the 100 ms on/50 ms off condition, which showed a similar power output. As the off-time increased, the calorimetric power decline compared to CW. Fig. 1a shows that as the off-time increases there is a steeper decline in calorimetric power with 10 ms on-times compared to the 100 ms on-times in DI water. Consistent with this trend, Fig. S3a shows that calorimetric power for the 100 ms on-time is higher compared to the 10 ms on-times for all on:off ratios except the 1 to 5 on:off ratio (10 ms on,50 ms off; 100 ms on, 500 ms off). This trend suggests that there are either fewer cavitating bubbles and/or cooler cavitating bubbles with the 10 ms on-times.

As observed in Fig. 1b and 1c, the calorimetric power decreased in the presence of surface-active compounds in a similar trend as in DI water. Surfactants stabilize bubbles against dissolution by decreasing the rate of diffusion of gases from the bubble [16], [39], suggesting their presence would allow more bubbles to survive the off-time resulting in comparatively higher calorimetry. However, Fig. 1b and 1c show that at 100  µM, bubble behavior does not differ with or without surfactant as the off–time increases. Henglein et al. 1989 [22] and 1990 [38] saw a logarithmic decrease in iodine production with an increase in off-time. Similarly, when the calorimetric powers are plotted against the off-times on a logarithmic scale, the slopes of the regression all fall within the standard error of one another, suggesting there is no significant difference between DI water (Fig. 1a) and FtS (Fig. 1b and 1c).

At a concentration of 100 µM, surface accumulation does not appear to play a role in stabilizing cavitation bubbles. The time scale for surfactants to reach saturation at the bubble interface is much longer than the lifetime of ultrasonically produced bubbles. The near equilibrium of dynamic surface adsorption for surfactants has been shown to be on the scale of a few to tens of milliseconds [40], [41], [42], [43]. The lifetime of a bubble produced by ultrasound, on the other hand, is thought to be on the scale of tens to hundreds of microseconds [44]. While off–times in PW systems may extend bubble lifetime, the increase is far too small relative to the much longer adsorption times required for surfactants. As a result, PW does not prolong bubble lifetime enough for surface activity to meaningfully influence bubble behavior. Although surface tension data shows there are more 8:2 FtS molecules accumulating at the bubble interfaces (Fig. S4), they do not stabilize the bubbles to affect sonochemical activity.

Using calorimetry as a dosimeter allows for direct observation of the energy released from cavitating bubbles. Comparing Fig. 1b and 1c to Fig. 1a, we observe lower calorimetry with FtS compared to DI water with CW. In PW systems and as off-time increases, the decrease in sonochemical activity is similar between DI and FtS. Evaluating semi volatile compounds at millimolar concentrations, de Visscher et al. 1996 [45] observed non-linear degradation kinetics and attributed the non-linearity to organic compounds in the bubble lowering the cavitation temperature. If energy from cavitating bubbles were diverted to breaking carbon–fluorine bonds, a decrease in power outputs in the presence of FtS (Fig. 1b and 1c) in comparison to DI water (Fig. 1a) would be expected. The decrease in calorimetry with FtS, even at CW, suggests that FtS is affecting heat released from cavitating bubbles, either by reducing the number of cavitating bubbles or reducing bubble temperatures. The lack of difference in sonochemical activity with increased off-time between DI and FtS indicates the reduction is independent of pulsing conditions and the time-scale of off-times is not enough to alter FtS bubble surface accumulation. Regardless, calorimetry may be used as a dosimeter to assess sonochemical activity and may even show reduced sonochemical activity due to compounds reacting.

Under all PW and surfactant parameters, there was a decrease in calorimetric power compared to CW except for the 100 ms on-time with the shortest off time. In DI water, in most cases, the 100 ms on-time has higher calorimetric output suggesting that 100 ms on-time provides more favorable bubble collapse than the 10 ms on-time. The similar decay in sonochemical activity observed for both DI water and FtS in Fig. 1 suggests that, within the off-time intervals investigated, FtS does not adsorb to the bubble surface during the off-time. A decrease in calorimetric power indicates a decrease in active bubbles or bubble temperature and therefore decreased sonochemical activity. The two mechanisms that control sonochemical activity in PW systems are bubble coalescence and dissolution. It is evident from the results presented in Fig. 1 that one or both mechanisms cause a drastic decrease in the calorimetric power.

### 3.2 Effects of PW and surfactant parameters on bubble coalescence

The capillary method following Lee et al. 2005 [28] was used to quantify bubble coalescence during ultrasound exposures with varying PW and surfactant conditions. This technique measures changes in total bubble volume during sonication, allowing quantification of degassing bubbles formed through coalescence. An increase in total bubble volume indicates an increase in bubble coalescence.

#### 3.2.1 Effect of PW parameters on bubble coalescence in DI water

**Fig. 2:**
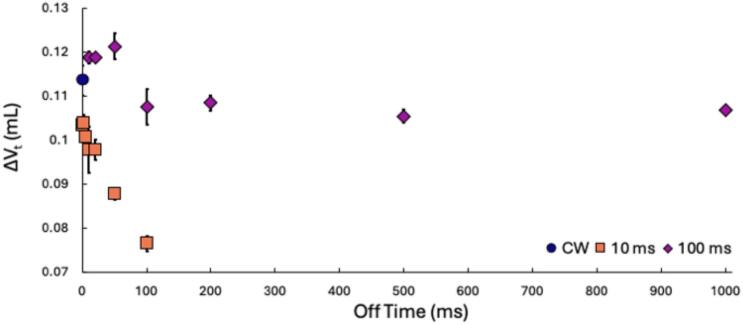
Changes in total bubble volume (ΔV_t_) under CW (blue circle), 10 ms on-time (orange square), and 100 ms on-time (magenta diamond) with on:off ratios from 1:0.5 to 1:10 in DI water at a frequency of 337 kHz. Error bars indicate standard error from triplicate experiments. In some cases, error bars are covered by the symbol.

Calorimetry results show a significant decrease in sonochemical activity as off–time increases. To determine whether this decrease arises from bubble coalescence or bubble dissolution, we examined bubble coalescence under PW conditions (Fig. 2; Table S8). In DI water, all off–times with a 10  ms on–time produced less coalescence than CW. For 100  ms on–times, coalescence was slightly higher than CW when the off–time was shorter than the on–time, but similar or slightly lower at off–times between 100 and 1000  ms.

Taken alone, the decrease in coalescence with longer off–times might suggest an increase in the number of active bubbles and thus greater sonochemical activity. However, our calorimetry results and previous dosimetry studies [22], [35], [38] consistently show the opposite trend: sonochemical activity decreases as off–time increases. Henglein et al. 1989 [22] observed a drastic decrease in luminescence intensity with increasing off-time, eventually approaching zero. They noted that shorter on-time pulses required more pulses to reach their maximum intensity. Similarly, Henglein et al. 1990 [38] and Gutierrez et al. 1990 [35] found that iodine yield and polymer degradation decreased as pulse off-time increased.

The coalescence results suggest that shorter on-times with longer off-times create more favorable conditions than longer on-times regarding bubble coalescence. If coalescence controls sonochemical activity, a decrease in bubble coalescence should correspond to more active bubbles in the system, and therefore more sonochemical activity. The results obtained from the capillary experiments, however, are in direct contrast with the calorimetry data presented in Section 3.1 that shows a decreased sonochemical activity with increased off-time.

#### 3.2.2 Effect of surfactant concentration and chain length on bubble coalescence

To understand if chemical compounds undergoing degradation may affect coalescence, we evaluated the role of three FtS compounds. Surfactants partition and adsorb to the air–water interface of the bubbles [46], [47]. Surfactants at bubble interfaces decrease coalescence through steric hindrance or electrostatic repulsive forces interfering with the contact time of bubbles and/or film thinning process during coalescence [27], [48]. Previous studies evaluated bubble coalescence at millimolar to molar concentration ranges, the ranges at which surface activity increased for the studied compounds [28], [29], [31], [49], [50], [51]. The concentrations chosen for this study correspond more closely to the concentration range reported for PFAS in environmental samples (μM range) [52], [53], [54], [55], [56], [57]. In addition, three different compounds of similar chemical composition were tested to determine the importance of surface activity vs concentration.

**Fig. 3:**
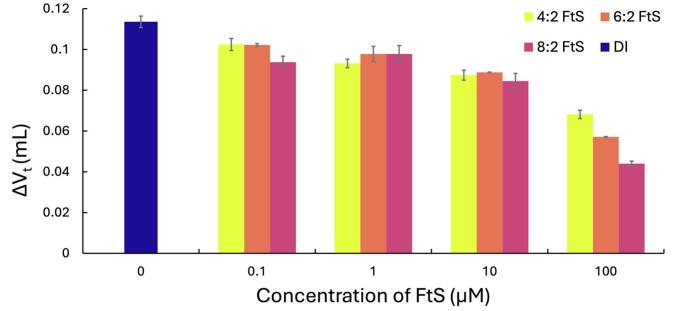
Effects of concentration on ΔV_t_ in 4:2, 6:2, and 8:2 FtS solutions under CW conditions (frequency: 337 kHz) compared to ΔV_t_ in DI water under CW conditions (frequency: 337 kHz). Error bars indicate standard error from triplicate experiments.

Fig. 3 and Table S9 in the SI show the change in total bubble volume (ΔV_t_) of solutions containing FtS under CW ultrasound. For all three FtS compounds, Fig. 3 shows that there was a decrease in bubble coalescence even at the lowest concentration of 0.1 μM. In addition, bubble coalescence decreased with an increase in surfactant concentration. The continued decrease in ΔV_t_ aligns with previous studies and theory that coalescence will decrease with increasing concentrations due to repulsive forces and steric hinderance [27], [28], [29], [49], [48].

As shown in Fig. 3, there was no difference between the three FtS until the highest concentration of 100 µM. At this concentration, 8:2 FtS shows a larger decrease in bubble coalescence compared to 6:2 and 4:2 FtS [58]. Surface tension data in Fig. S4 shows that 8:2 FtS accumulates at the bubble surface more than 6:2 and 4:2 FtS at the same concentration, which leads to decreased bubble coalescence for 8:2 FtS. An increase in the number of carbons in the alkyl chain increases the hydrophobicity of the surfactant, increasing its ability to accumulate at a bubble surface. Lee et al. 2005 [28] measured bubble coalescence in the presence of charged surfactants of varying surface activities but did not find any noticeable trends concerning changes in surface activity. Their results were unexpected because compounds with higher surface activity should decrease bubble coalescence. Consistent with the trend of surface activity, our study used compounds that only differed by chain lengths to allow for more direct comparison of surface activity of the surfactant.

Fig. 3 shows that concentrations as low as 0.1 µM FtS decreased bubble coalescence. However, Fig. S4 shows that there is no difference in surface tension for 4:2 FtS in comparison to DI water; even at the highest concentration of 100 µM. There is a slight decrease in surface tension for 6:2 FtS at 100 µM. Ashokkumar et al. 1997 [30] found that the addition of surfactant to aqueous solutions decreases the density of the bubble cloud where sonochemical activity occurs. They speculated that decreasing the density of the bubble cloud led to decreased collisions and contact times of bubbles, therefore decreasing the rate of bubble coalescence. Therefore, while surface activity plays a role at concentrations that impact the surface tension, bubble coalescence is affected even at low concentrations of FtS such as 0.1 µM by “declustering” the bubble cloud.

#### 3.2.3 Combined PW and surfactant effects on bubble coalescence

**Fig. 4:**
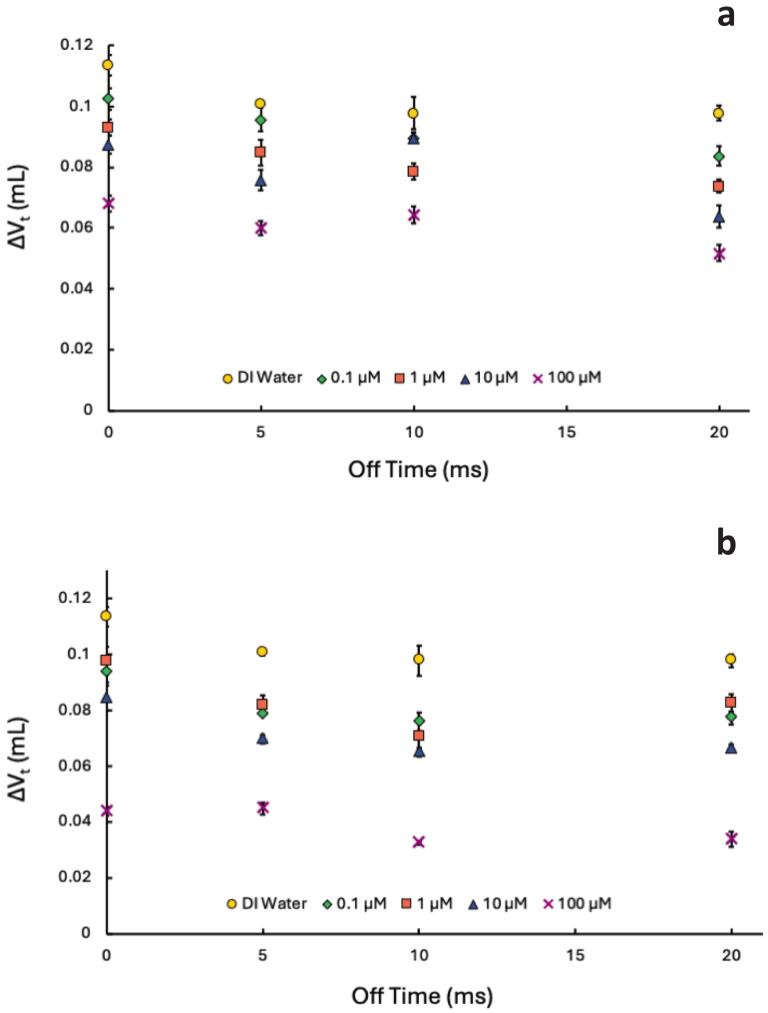
ΔV_t_ at various off-times with an on-time of 10 ms in solutions of a) 4:2 FtS and b) 8:2 Fts (frequency: 337 kHz). The 0 ms off-time corresponds to CW experiments. Concentrations are represented by different symbol shapes and colors: DI Water (0 μM) = yellow circles, 0.1 μM = green diamonds, 1 μM = orange squares, 10 μM = blue diamonds, 100 μM = purple Xs. Error bars indicate standard error from triplicate experiments.

Next, we examined the effects of FtS under PW conditions to determine whether combining PW operation with surfactants further reduces bubble coalescence. Fig. 4 and Table S10 show a small decrease in bubble coalescence for the 10 ms on-time for both the 4:2 and 8:2 FtS as the off-time increased to 20 ms. The most pronounced and most consistent difference between PW and CW conditions occurs at this longest off-time for both FtS. For example, for experiments in 100 μM 4:2 FtS (purple Xs Fig. 4a), ΔV_t_ decreases from 0.068 ± 0.003 to 0.052 ± 0.003 under PW.

**Fig. 5:**
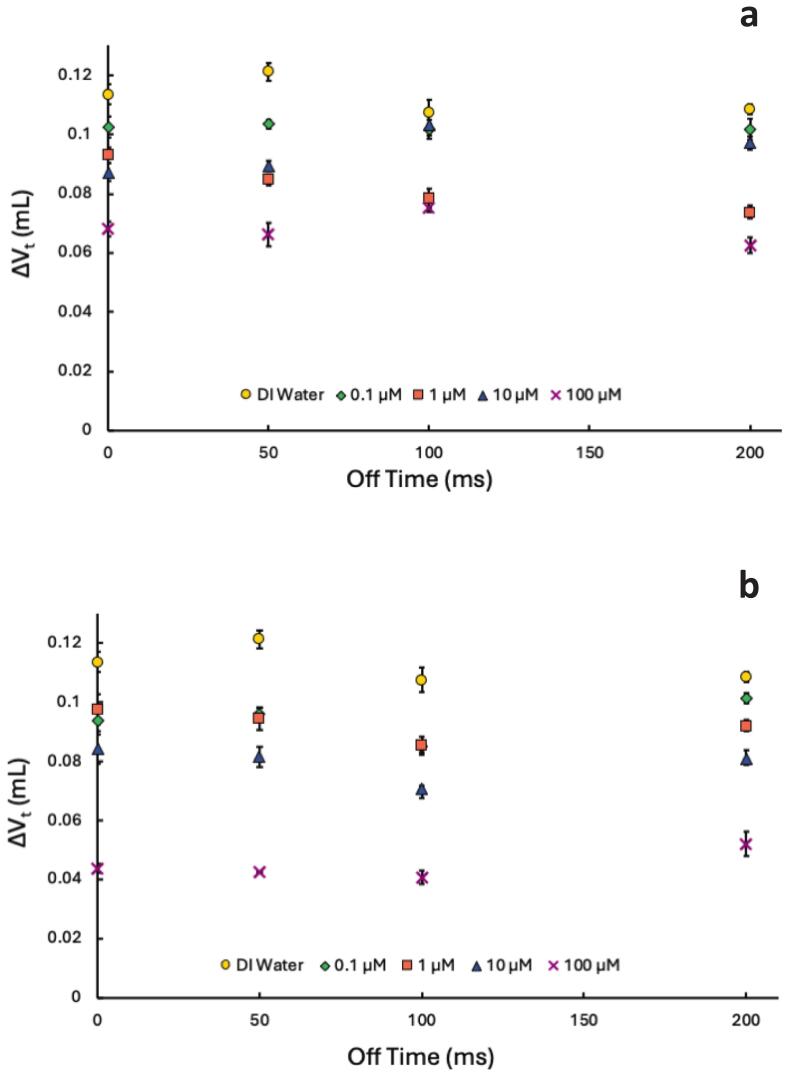
ΔV_t_ at various off-times with an on-time of 100 ms in solutions of a) 4:2 FtS and b) 8:2 Fts (frequency: 337 kHz). The 0 ms off-time corresponds to CW experiments. Concentrations are represented by different symbol shapes and colors: DI Water (0 μM) = yellow circles, 0.1 μM = green diamonds, 1 μM = orange squares, 10 μM = blue diamonds, 100 μM = purple Xs. Error bars indicate standard error from triplicate experiments.

In addition, Fig. 5 and Table S11 show even less difference between PW and CW with an on-time of 100 ms for both 4:2 and 8:2 FtS. Based on Fig. 3 in CW ultrasound, the more hydrophobic and surface-active compound, 8:2 FtS, reduces bubble coalescence more than 4:2 FtS. Thus, we expect PW conditions to amplify these effects. Longer off-times provide additional time for surface-active species to accumulate at the bubble interface, leading to greater reductions in coalescence than for less surface-active species and under CW conditions. [[16], [19], [59]].

In addition, studies of sonoluminescence [29], [31], [49], [50], inertial cavitation [29], [32], [33], [34], and degradation reactions [16], [38] found that longer on-times yielded higher reaction efficiencies than shorter on-times. These studies concluded that shorter pulse on-times caused increased rates of bubble coalescence, resulting in lower sonochemical activity. The results from Fig. 4, Fig. 5 suggest that in general, the 10 ms on-times provide more favorable conditions than the 100 ms on-time due to a decrease in bubble coalescence. For both 10 and 100 ms experiments, there were no significant trends across PW conditions. A lack of trends with off-times and surface activity does not align with previous studies that attributed increased degradation rates to longer off-times allowing more time for surfactants to accumulate at bubble interfaces. [[16], [17], [18], [60]].

While the results suggest 8:2 FtS partitions to the bubbles’ surface more than 4:2 FtS, the lack of trends suggest that there is no difference in coalescence in relation to varying off-times. Coalescence results suggest that while coalescence does decrease due to the presence of surface-active compounds (Fig. 3), there is minimal effect on bubble coalescence in PW systems in comparison to CW systems (Fig. 4, Fig. 5). The timescale of the off-times here (ms) to the timescale of off-times that observed significant pulse enhancement (s) [16] may be why we did not observe an effect on coalescence in PW with FtS. Overall, our results are counter to previous work [[2], [18], [22], [29], [35], [38], [59]] that suggest reduced coalescence plays an equal or more prominent role in controlling the sonochemical effectiveness in PW systems. In addition, there is a clear trend from the calorimetry data in Fig. 1 which, taken together with coalescence data, suggests that bubble dissolution, rather than coalescence, is the driving force in PW systems.

### 3.3 2-Hydroxytereplthalate (HTA) fluorescence measurements in PW systems

Our coalescence and dissolution data presented in this study appear to conflict. The calorimetry data in Fig. 1 shows a decrease in sonochemical activity for all PW conditions in the presence and absence of surfactants. The coalescence data indicates a decrease in coalescence in DI water with PW (Fig. 2) and with FtS with CW (Fig. 3), but minimal added effects in PW systems in the presence of surfactants (Fig. 4, Fig. 5). Hydroxyl radical formation using the HTA dosimeter was used to validate calorimetry as a simple dosimeter. The initial zero-order rate constant of HTA production was determined for the two pulse on-times at the various off-times and compared to the initial rate constant of HTA for CW systems. Higher initial rate constants of HTA production correspond to higher production of hydroxyl radicals and therefore more and/or hotter cavitating bubbles.

**Fig. 6:**
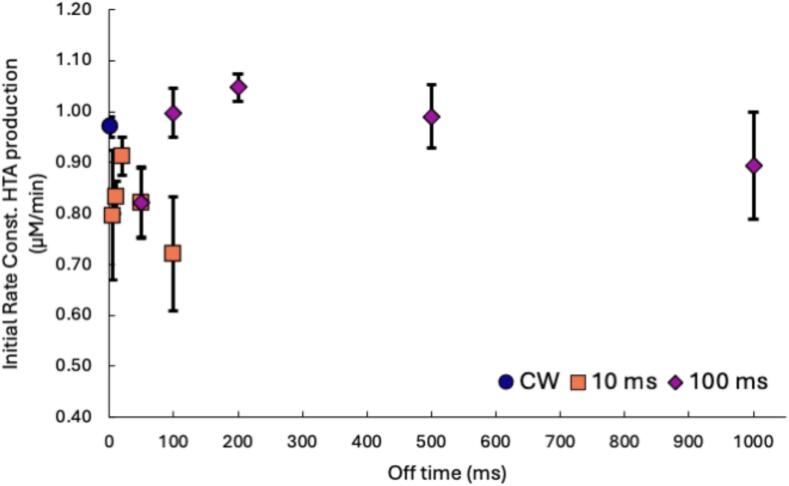
Initial rate constants of HTA production under CW (blue circle), 10 ms on-time (orange square), and 100 ms on-time (purple diamond) with on:off ratios from 1:0.5 to 1:10 in 1 mM terephthalic acid solutions. Error bars indicate standard error from triplicate experiments.

Fig. 6 and Table S12 in the SI show a similar trend to the calorimetry data in Fig. 1. As the off-time increases, there is a significant decrease in sonochemical activity for both the 10 and 100 ms on-times as measured by initial rate constants of HTA formation. The drop in the production rate of HTA formation indicates a decrease in the hydroxyl radicals produced from cavitation bubbles. All the HTA production rates for the 100 ms on-time were higher than the 10 ms on-time. This trend is consistent with the calorimetry data, which showed higher calorimetric power for all but one of the 100  ms on–time experiments compared with the 10  ms on–time at the same on:off ratios. Fig. 6 also shows a slight increase in HTA formation in some of the PW experiments in comparison to CW with an on-time of 100 ms. Tuziuti et al. 2008 [21] and Lee et al. 2008 [20] observed that the spatial distribution of the active zone for cavitation increases in PW fields. This increase in the active zone leads to a higher percentage of standing waves in the acoustic field, decreasing the production of degassing bubbles from coalescence. Degassing bubbles decrease the number of active bubbles in solution. Therefore, decreasing the formation of degassing bubbles leads to higher sonochemical efficiency. While it is possible a similar mechanism is at play in our system, the volumes used in our study are half that of the smallest volume studied in Tuziuti et al. 2008. Further experiments are necessary to establish the role a change in the acoustic field is playing between the PW and CW fields.

## 4 Implications and Conclusion

The calorimetry and hydroxyl radical production both indicate a decrease in the number of cavitating bubbles or decreased bubble temperatures during bubble collapse with an increase in off-time. This trend is opposite to the coalescence data which showed a decrease in bubble coalescence suggesting an increase in cavitating bubbles under PW conditions. The effect is most pronounced with the 10 ms on-times as the off-time increased in DI water (Fig. 2). In addition, whether in DI water (Fig. 2) or in FtS (Fig. 4, Fig. 5), the 100 ms on-time showed minimal to no changes in bubble coalescence in comparison to CW.

The coalescence data also suggests the 10 ms on-time leads to more favorable conditions by minimizing bubble coalescence. However, the calorimetry and HTA formation results indicate more sonochemical activity with 100 ms on-time. Both coalescence and dissolution affect the sonochemical activity in PW systems. While previous work indicates increased coalescence leads to a decrease in sonochemical activity, there is less coalescence during the off-times without the driving force of Bjerknes forces. In addition, if the on-time is too short, bubbles do not have enough time to grow to a size that can withstand dissolution due to the Laplace pressure [61]. When the off-time is too long, there is a higher likelihood that bubbles will dissolve due to the Laplace pressure [[10], [29], [32], [62]]. If the bubble has not grown to a size large enough to withstand the Laplace pressure, the bubbles will decay (*i.e.*, dissolve) to a point where they need to be reactivated by the next pulse [[29], [30], [32], [62], [63]]. In the case of the 10 ms on-times, dissolution is out-pacing coalescence leading to decreased ΔV_t_ and decreased sonochemical activity.

Longer on-times producing more sonochemical activity is consistent with previous work with PW systems. In pulsed systems with a short on-time, the system takes longer to activate due to shorter acoustic cycles which causes fewer bubbles to survive the off period [22], [30]. Ashokkumar et al. 1997 [30] and Tronson et al. 2002 [31] found that sonoluminescence in PW systems increased with longer on-times. Inertial cavitation has also been used to monitor bubble activity in PW systems, and these studies have found that cavitation activity increases with longer on-times. [[29], [32], [33], [34], [62]].

Synthesizing results from the three methods, it appears that bubble dissolution is the controlling factor in PW systems rather than reduced coalescence. Coalescence in an ultrasound field is driven by the primary and secondary Bjerknes forces which are, respectively, the forces that push bubbles to the nodes/antinodes and attract neighboring oscillating bubbles to one another. These forces result in bubbles of similar size and in-phase to coalesce, thus decreasing the number of active bubbles. [10], [26] While it is possible coalescence still occurs in PW systems, it is unlikely to be more prominent than in CW systems. The introduction of off-times in PW systems reduces coalescence due to the lack of Bjerknes forces. Instead, bubble dissolution determines the efficiency of pulsed wave systems. Bubble growth is halted during the off periods due to the absence of ultrasound waves. Bubbles that are too small cannot withstand the Laplace pressure within the bubble and will dissolve, decreasing the number of active bubbles. Extended off-times can, therefore, increase the rates of bubble dissolution, which is consistent with calorimetry trends in Fig. 1 and HTA trends in Fig. 6. Therefore, the decrease in power and HTA production is due to there being fewer cavitating bubbles due to increased rates of bubble dissolution. While coalescence occurs under these parameters, it is not the driving mechanism that controls sonochemical activity in PW systems.

This study evaluated whether bubble coalescence or dissolution played a more prominent role in the effectiveness of PW systems. We used calorimetry as a dosimeter for sonochemical activity. The results from the calorimetry experiments suggest that there is a significant decrease in sonochemical activity in PW systems as the off-time increases. These results are supported by a decrease in HTA formation with an increase in off-time. This suggests that calorimetry can be used as a quicker and easier dosimeter, that requires no additional chemicals, to assess the sonochemical activity of PW systems. In addition, the calorimetry and terephthalic acid methods suggest the longer on-time of 100 ms with shorter off-times create a more favorable environment for sonochemical activity. While the coalescence data suggests the 10 ms on-time is the more favorable pulse on-time by decreasing coalescence, the increased bubble dissolution controls sonochemical activity. Both calorimetry and HTA results are more aligned with previous literature that shows decreased sonochemical activity at shorter on-time and as the off-time increases.

Despite the observed decline in sonochemical activity under PW conditions in our study, PW ultrasound remains a potentially relevant tool. While our results demonstrate that bubble dissolution limits overall hydroxyl radical production and calorimetric power in PW systems, these metrics primarily reflect bulk-phase activity and may not fully capture the degradation potential for highly surface-active or volatile contaminants. The introduction of off-times increase the effective lifetime of bubbles, potentially providing surface-active compounds, such as PFAS, additional time to partition to the bubble interface. If the amount of surfactant at the interface is increased by these pulse intervals, the resulting increase in local concentration could offset the reduction in total cavitating bubbles. Therefore, PW ultrasound may be optimized for specific complex mixtures where the kinetic benefit of surface partitioning outweighs the losses driven by bubble dissolution.

## CRediT authorship contribution statement

**Marc C. Nolan:** Writing – original draft, Visualization, Methodology, Investigation, Data curation, Conceptualization. **Haleigh A. Fernandez:** Writing – review & editing, Methodology, Investigation, Conceptualization. **Amanda N. Cowen:** Writing – review & editing, Methodology, Investigation. **Fallon R. Fuller:** Writing – review & editing, Methodology, Investigation. **Linda K. Weavers:** Writing – review & editing, Supervision, Project administration, Funding acquisition, Conceptualization.

## Declaration of competing interest

The authors declare that they have no known competing financial interests or personal relationships that could have appeared to influence the work reported in this paper.
