# Supplementary material for: Bubble coalescence and dissolution effects on sonochemical activity in pulsed wave ultrasound systems
Source: Ultrason Sonochem. 2026 Feb 16;127:107787. doi: 10.1016/j.ultsonch.2026.107787 (PMC12945590; doi:10.1016/j.ultsonch.2026.107787)
Supplement: Supplementary Data 1 [file mmc1.docx]

**Supplementary Information**

Bubble Coalescence and Dissolution Effects on Sonochemical Activity in Pulsed Wave Ultrasound Systems

Marc C. Nolan^1,2^, Haleigh A. Fernandez^1,2,3^, Amanda N. Cowen^2^, Fallon R. Fuller^2^, Linda K. Weavers^1,2,3^

^1^Environmental Sciences Graduate Program, The Ohio State University, Columbus, OH 43210

^2^Department of Civil, Environmental, and Geodetic Engineering, The Ohio State University, Columbus, OH 43210

^3^Ohio Water Resources Center, The Ohio State University, Columbus, OH 43210

**This Supplementary Information contains 12 tables and 4 figures as described in the table of contents.**

**Table of Contents**

**Table S1.** Calorimetric power (W) under CW conditions in DI water and 100 μM of 4:2 and 8:2 FtS in air and argon sparged solutions.

**Table S2.** Change in temperature during CW and PW calorimetry experiments in DI water. The temperatures represent the average change in temperature of the glass chamber that surrounds the reaction vessel (collected by the type T thermocouple).

**Table S3.** Change in temperature during continuous wave coalescence experiments in DI water.

**Table S4.** The effect of pH on changes in total bubble volume (∆V_t_) during coalescence experiments.

**Table S5.** Average calorimetric power (W) under CW and PW conditions in DI water.

**Table S6.** Average calorimetric power (W) under CW and PW conditions in 100 µM 4:2 FtS.

**Table S7.** Average calorimetric power (W) under CW and PW conditions in 100 µM 8:2 FtS.

**Table S8.** Average change in total bubble volume (∆V_t_) from coalescence experiments in DI water.

**Table S9.** Average change in total bubble volume (∆V_t_) from coalescence experiments under CW conditions.

**Table S10.** Average change in total bubble volume (∆V_t_) from coalescence experiments in the presence of 4:2 and 8:2 FtS at various concentrations. The 0 µM experiments correspond to PW conditions in DI water.

**Table S11.** Average change in total bubble volume (∆V_t_) from coalescence experiments in the presence of 4:2 FtS at various concentrations. The 0 µM experiments correspond to PW conditions in DI water.

**Table S12.** Average initial rate constants of HTA production from sonication of 1mM terephthalic acid oxidation solutions under CW and PW conditions.

**Figure S1.** Experimental setup for the calorimetry experiments. The type K thermocouple on the left monitored the change in temperature of the solution. The type T thermocouple on the right monitored the change in temperature of the glass chamber that housed the solution.

**Figure S2.** Capillary system for measuring change in total bubble volume (∆V_t_)

**Figure S3.** Results from calorimetry experiments under CW and PI conditions in a) DI water, b) 100 µM 4:2 FtS, and c) 100 µM 8:2 FtS.

**Figure S4.** Average surface tensions measurements of DI water, 4:2 FtS, 6:2 FtS, and 8:2 FtS solutions at various concentrations.


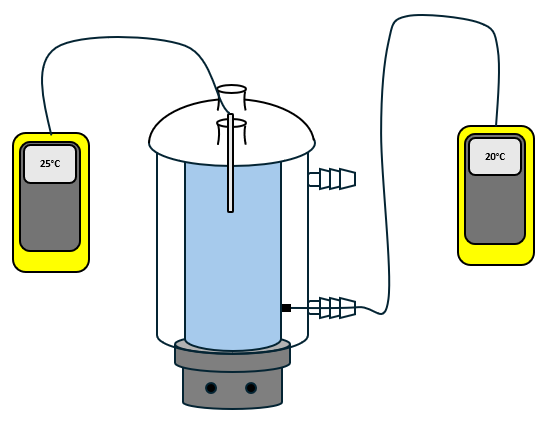


**Figure S1.** Experimental setup for the calorimetry experiments. The type K thermocouple on the left monitored the change in temperature of the solution. The type T thermocouple on the right monitored the change in temperature of the glass chamber that housed the solution.

The temperature measurements from the type T thermocouple were used to determine the amount of heat that escapes the solution during CW and PW experiments. The change in temperature from CW experiments was used as a baseline for heat lost to the surroundings. The heat lost during the PW experiments was then compared to CW experiments. Results shown in Table S1 below.

**Table S1.** Calorimetric power (W) under CW conditions in DI water and 100 μM of 4:2 and 8:2 FtS in air and argon sparged solutions.

| US Condition | Solution Type | Gas Conditions | Avg Calorimetric Power (W) |
| --- | --- | --- | --- |
| CW | DI water | Air | 43.3 ± 0.2 |
| CW | DI water | Argon | 42.6 ± 0.6 |
| CW | 4:2 FtS | Air | 40.4 ± 0.3 |
| CW | 4:2 FtS | Argon | 40.47 ± 0.08 |
| CW | 8:2 FtS | Air | 41.6 ± 0.4 |
| CW | 8:2 FtS | Argon | 41.20 ± 0.10 |

**Table S2.** Change in temperature during CW and PW calorimetry experiments in DI water. The temperatures represent the average change in temperature of the glass chamber that surrounds the reaction vessel (collected by the type T thermocouple). Error terms are standard error collected from triplicate experiments.

| US Condition | Pulse on-time (ms) | Pulse off-time (ms) | Avg Temp of type-T multimeter (°C) |
| --- | --- | --- | --- |
| CW | n/a | n/a | 4.3 ± 0.3 |
| PW | 10 | 5 | 4.7 ± 0.3 |
| PW | 10 | 10 | 4.2 ± 0.2 |
| PW | 10 | 20 | 4.2 ± 0.6 |
| PW | 100 | 50 | 4.6 ± 0.4 |
| PW | 100 | 100 | 4.9 ± 0.7 |
| PW | 100 | 200 | 4.5 ± 0.3 |


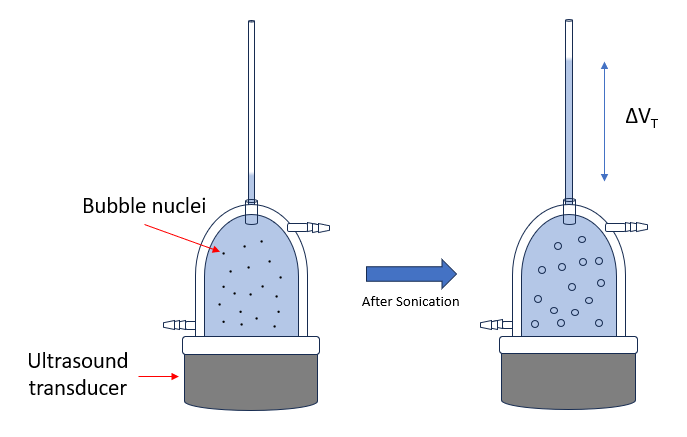


**Figure S2.** Capillary system for measuring change in total bubble volume (∆V_t_)

**Table S3.** Change in temperature during continuous wave coalescence experiments in DI water.

| Initial Temperature (°C) | Final Temperature (°C) | Change in Temperature (°C) |
| --- | --- | --- |
| 21.5 | 22.0 | 0.5 |
| 21.1 | 21.6 | 0.5 |
| 21.2 | 21.8 | 0.6 |

**Table S4.** The effect of pH on changes in total bubble volume (∆V_t_) during coalescence experiments. Error terms are standard error collected from triplicate experiments.

| pH | ∆Vt (mL) |
| --- | --- |
| 3.5 | 0.108 ± 0.006 |
| 6.5 | 0.1102 ± 0.0014 |
| 9.5 | 0.114 ± 0.003 |

**Table S5.** Average calorimetric power (W) under CW and PW conditions in DI water. Error terms are standard error collected from triplicate experiments.

| US Condition | On Time (ms) | Off Time (ms) | On:Off Ratio | Avg Power (W) |
| --- | --- | --- | --- | --- |
| CW | n/a | n/a | n/a | 43.3 ± 0.2 |
| PW | 10 | 5 | 1 to 0.5 | 40.5 ± 0.2 |
| PW |  | 10 | 1 to 1 | 38.9 ± 0.2 |
| PW |  | 20 | 1 to 2 | 36.1 ± 0.6 |
| PW |  | 50 | 1 to 5 | 34.2 ± 0.6 |
| PW |  | 100 | 1 to 10 | 26.1 ± 0.5 |
| PW | 100 | 50 | 1 to 0.5 | 42.8 ± 0.6 |
| PW |  | 100 | 1 to 1 | 39.6 ± 0.2 |
| PW |  | 200 | 1 to 2 | 38.4 ± 0.2 |
| PW |  | 500 | 1 to 5 | 32.8 ± 0.2 |
| PW |  | 1000 | 1 to 10 | 29.0 ± 0.3 |

**Table S6.** Average calorimetric power (W) under CW and PW conditions in 100 µM 4:2 FtS. Error terms are standard error collected from triplicate experiments.

| US Condition | On Time (ms) | Off Time (ms) | On:Off Ratio | Avg Power (W) |
| --- | --- | --- | --- | --- |
| CW | n/a | n/a | n/a | 40.4 ± 0.3 |
| PW | 10 | 5 | 1 to 0.5 | 39.5 ± 0.3 |
| PW |  | 10 | 1 to 1 | 37.9 ± 0.3 |
| PW |  | 20 | 1 to 2 | 35.4 ± 0.6 |
| PW |  | 50 | 1 to 5 | 31.6 ± 0.12 |
| PW |  | 100 | 1 to 10 | 27.7 ± 0.4 |
| PW | 100 | 50 | 1 to 0.5 | 41.7 ± 0.4 |
| PW |  | 100 | 1 to 1 | 39.2 ± 0.3 |
| PW |  | 200 | 1 to 2 | 38.1 ± 0.10 |
| PW |  | 500 | 1 to 5 | 32.4 ± 0.3 |
| PW |  | 1000 | 1 to 10 | 27.7 ± 0.4 |

**Table S7.** Average calorimetric power (W) under CW and PW conditions in 100 µM 8:2 FtS. Error terms are standard error collected from triplicate experiments.

| US Condition | On Time (ms) | Off Time (ms) | On:Off Ratio | Avg Power (W) |
| --- | --- | --- | --- | --- |
| CW | n/a | n/a | n/a | 41.6 ± 0.4 |
| PW | 10 | 5 | 1 to 0.5 | 40.5 ± 0.14 |
| PW |  | 10 | 1 to 1 | 38.1 ± 0.2 |
| PW |  | 20 | 1 to 2 | 36.5 ± 0.02 |
| PW |  | 50 | 1 to 5 | 32.5 ± 0.07 |
| PW |  | 100 | 1 to 10 | 27.0 ± 0.8 |
| PW | 100 | 50 | 1 to 0.5 | 41.1 ± 0.4 |
| PW |  | 100 | 1 to 1 | 39.6 ± 0.3 |
| PW |  | 200 | 1 to 2 | 36.4 ± 0.5 |
| PW |  | 500 | 1 to 5 | 33.2 ± 0.4 |
| PW |  | 1000 | 1 to 10 | 27.6 ± 0.4 |

**a**

**b**

**c**

**Figure S3.** Results from calorimetry experiments under CW and PI conditions in a) DI water, b) 100 µM 4:2 FtS, and c) 100 µM 8:2 FtS. Error bars are standard error collected from triplicate experiments.

**
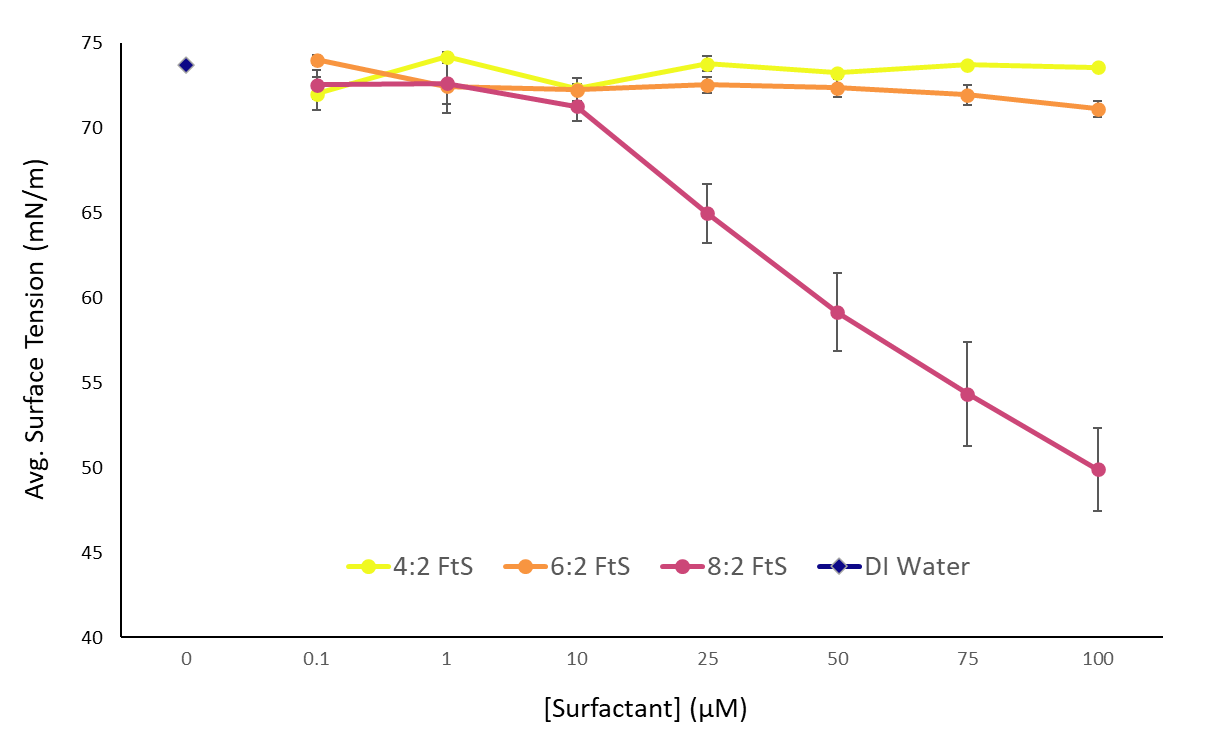
**

**Figure S4.** Average surface tensions measurements of DI water, 4:2 FtS, 6:2 FtS, and 8:2 FtS solutions at various concentrations. The procedure described in Fagan et al. 2023 was used to obtained these data. The error bars represent that standard deviation from triplicate trials.

**Table S8.** Average change in total bubble volume (∆V_t_) from coalescence experiments in DI water. Error terms are standard error collected from triplicate experiments.

| US Condition | On-time (ms) | Off-time (ms) | On:Off Ratio | Avg ∆V_t_  (mL) |
| --- | --- | --- | --- | --- |
| CW | n/a | n/a | n/a | 0.114 ± 0.003 |
| PW | 10 | 1 | 1 to 0.1 | 0.1034 ± 0.0003 |
|  |  | 2 | 1 to 0.2 | 0.104 ± 0.002 |
|  |  | 5 | 1 to 0.5 | 0.101 ± 0.0011 |
|  |  | 10 | 1 to 1 | 0.098 ± 0.005 |
|  |  | 20 | 1 to 2 | 0.098 ± 0.002 |
|  |  | 50 | 1 to 5 | 0.088 ± 0.0013 |
|  |  | 100 | 1 to 10 | 0.077 ± 0.002 |
|  | 100 | 10 | 1 to 0.1 | 0.119 ± 0.0013 |
|  |  | 20 | 1 to 0.2 | 0.1187 ± 0.0009 |
|  |  | 50 | 1 to 0.5 | 0.121 ± 0.003 |
|  |  | 100 | 1 to 1 | 0.108 ± 0.004 |
|  |  | 200 | 1 to 2 | 0.108 ± 0.002 |
|  |  | 500 | 1 to 5 | 0.105 ± 0.0014 |
|  |  | 1000 | 1 to 10 | 0.1069 ± 0.0008 |

**Table S9.** Average change in total bubble volume (∆V_t_) from coalescence experiments under CW conditions. Error terms are standard error collected from triplicate experiments.

| Solution Type | Concentration (µM) | Avg ∆V_t_  (mL) |
| --- | --- | --- |
| DI water | 0 | 0.114 ± 0.003 |
| 8:2 FtS | 0.1 | 0.094 ± 0.004 |
|  | 1 | 0.098 ± 0.005 |
|  | 10 | 0.084 ± 0.005 |
|  | 100 | 0.044 ± 0.002 |
| 4:2 FtS | 0.1 | 0.102 ± 0.004 |
|  | 1 | 0.093 ± 0.003 |
|  | 10 | 0.087 ± 0.003 |
|  | 100 | 0.068 ± 0.003 |

**Table S10.** Average change in total bubble volume (∆V_t_) from coalescence experiments in the presence of 4:2 and 8:2 FtS at various concentrations. The 0 µM experiments correspond to PW conditions in DI water. Error terms are standard error collected from triplicate experiments.

| FtS Type | On-time (ms) | Off-time (ms) | On:Off Ratio | Concentration (µM) | Avg ∆V_t_  (mL) |
| --- | --- | --- | --- | --- | --- |
| 4:2 | 10 | 5 | 1 to 0.5 | 0 | 0.101 ± 0.0011 |
|  |  |  | 5 | 0.1 | 0.096 ± 0.004 |
|  |  |  |  | 1 | 0.085 ± 0.004 |
|  |  |  |  | 10 | 0.076 ± 0.003 |
|  |  |  |  | 100 | 0.060 ± 0.002 |
|  |  | 10 | 1 to 1 | 0 | 0.098 ± 0.005 |
|  |  |  |  | 0.1 | 0.090 ± 0.002 |
|  |  |  |  | 1 | 0.079 ± 0.003 |
|  |  |  |  | 10 | 0.090 ± 0.002 |
|  |  |  |  | 100 | 0.064 ± 0.003 |
|  |  | 20 | 1 to 2 | 0 | 0.098 ± 0.002 |
|  |  |  |  | 0.1 | 0.084 ± 0.003 |
|  |  |  |  | 1 | 0.074 ± 0.002 |
|  |  |  |  | 10 | 0.064 ± 0.004 |
|  |  |  |  | 100 | 0.052 ± 0.003 |
| 8:2 | 10 | 5 | 1 to 0.5 | 0 | 0.1007 ± 0.0011 |
|  |  |  |  | 0.1 | 0.0787 ± 0.0002 |
|  |  |  |  | 1 | 0.082 ± 0.004 |
|  |  |  |  | 10 | 0.070 ± 0.0015 |
|  |  |  |  | 100 | 0.045 ± 0.002 |
|  |  | 10 | 1 to 1 | 0 | 0.098 ± 0.005 |
|  |  |  |  | 0.1 | 0.076 ± 0.003 |
|  |  |  |  | 1 | 0.071 ± 0.005 |
|  |  |  |  | 10 | 0.065 ± 0.002 |
|  |  |  |  | 100 | 0.0326 ± 0.0005 |
|  |  | 20 | 1 to 2 | 0 | 0.098 ± 0.002 |
|  |  |  |  | 0.1 | 0.077 ± 0.002 |
|  |  |  |  | 1 | 0.083 ± 0.003 |
|  |  |  |  | 10 | 0.066 ± 0.0014 |
|  |  |  |  | 100 | 0.034 ± 0.003 |

**Table S11.** Average change in total bubble volume (∆V_t_) from coalescence experiments in the presence of 4:2 FtS at various concentrations. The 0 µM experiments correspond to PW conditions in DI water. Error terms are standard error collected from triplicate experiments.

| FtS Type | On-time (ms) | Off-time (ms) | On:Off Ratio | Concentration (µM) | Avg ∆V_t_  (mL) |
| --- | --- | --- | --- | --- | --- |
| 4:2 | 100 | 50 | 1 to 0.5 | 0 | 0.121 ± 0.003 |
|  |  |  |  | 0.1 | 0.104 ± 0.0014 |
|  |  |  |  | 1 | 0.094 ± 0.002 |
|  |  |  |  | 10 | 0.089 ± 0.002 |
|  |  |  |  | 100 | 0.066 ± 0.004 |
|  |  | 100 | 1 to 1 | 0 | 0.108 ± 0.004 |
|  |  |  |  | 0.1 | 0.102 ± 0.003 |
|  |  |  |  | 1 | 0.106 ± 0.003 |
|  |  |  |  | 10 | 0.103 ± 0.004 |
|  |  |  |  | 100 | 0.076 ± 0.002 |
|  |  | 200 | 1 to 2 | 0 | 0.108 ± 0.002 |
|  |  |  |  | 0.1 | 0.102 ± 0.004 |
|  |  |  |  | 1 | 0.099 ± 0.002 |
|  |  |  |  | 10 | 0.097 ± 0.002 |
|  |  |  |  | 100 | 0.063 ± 0.003 |
| 8:2 | 100 | 50 | 1 to 0.5 | 0 | 0.121 ± 0.003 |
|  |  |  |  | 0.1 | 0.096 ± 0.002 |
|  |  |  |  | 1 | 0.094 ± 0.004 |
|  |  |  |  | 10 | 0.082 ± 0.003 |
|  |  |  |  | 100 | 0.04275 ± 0.0007 |
|  |  | 100 | 1 to 1 | 0 | 0.108 ± 0.004 |
|  |  |  |  | 0.1 | 0.085 ± 0.002 |
|  |  |  |  | 1 | 0.085 ± 0.003 |
|  |  |  |  | 10 | 0.071 ± 0.0012 |
|  |  |  |  | 100 | 0.041 ± 0.002 |
|  |  | 200 | 1 to 2 | 0 | 0.108 ± 0.002 |
|  |  |  |  | 0.1 | 0.101 ± 0.002 |
|  |  |  |  | 1 | 0.092 ± 0.002 |
|  |  |  |  | 10 | 0.081 ± 0.003 |
|  |  |  |  | 100 | 0.052 ± 0.004 |

**Table S12.** Average initial rate constants of HTA production from sonication of 1mM terephthalic acid oxidation solutions under CW and PW conditions. Error terms represent standard error based on triplicate experiments

| US condition | On time (ms) | Off time (ms) | Initial Rate Const. HTA (µM/min) |
| --- | --- | --- | --- |
| CW | n/a | n/a | 0.97 ± 0.02 |
| PW | 10 | 5 | 0.80 ± 0.13 |
| PW |  | 10 | 0.83 ± 0.03 |
| PW |  | 20 | 0.91 ± 0.04 |
| PW |  | 50 | 0.82 ± 0.07 |
| PW |  | 100 | 0.72 ± 0011 |
| PW | 100 | 50 | 0.82 ± 0.07 |
| PW |  | 100 | 1.00 ± 0.05 |
| PW |  | 200 | 1.05 ± 0.03 |
| PW |  | 500 | 0.99 ± 0.06 |
| PW |  | 1000 | 0.89 ± 0.11 |
